# Cultivating victory: workplace flourishing as a predictor of task performance in the Ghana Armed Forces

**DOI:** 10.3389/fpsyg.2025.1691941

**Published:** 2025-12-01

**Authors:** Theresa Esi Bosomtwe, Saviour Ayertey Nubuor

**Affiliations:** 1 Department of Human Resource Management, Knutsford University, Accra, Ghana; 2 Department of Organisation and Human Resource Management, University of Ghana, Accra, Ghana

**Keywords:** workplace flourishing, flourishing at work, task performance, Ghana armed forces, Social Exchange Theory (SET), Job Demand-Resource model (JD-R)

## Abstract

Military personnel in high-stress environments face significant risks to their well-being, which can impair their crucial task performance. This study investigated the relationship between workplace flourishing and task performance within the Ghana Armed Forces. Using a quantitative cross-sectional design, data were collected from 292 military personnel (from Second Lieutenant to Major and NCOs) stationed at the 37 Military Hospital via standardized scales: the Flourishing at Work Scale and the In-Role Performance Scale. The results revealed a significant positive correlation (*r* = 0.486, *p* < 0.01) and a predictive relationship (*β* = 0.339, *p* < 0.01), indicating that personnel who experience higher levels of holistic well-being—encompassing emotional, psychological, and social dimensions—demonstrate markedly better task performance. The findings underscore that fostering a flourishing work environment is a critical strategic component for enhancing operational effectiveness in military organizations.

## 1 Introduction

The United Nations Development Program (UNDP) emphasizes employee well-being as essential for achieving organizational goals, yet maintaining it remains a formidable challenge in today’s demanding business climate. Organizations, pressured by economic forces, often push employees to do more with less, increasing workloads and stress (Shukur and Kanona, 2021; Hunsaker, 2020). Gallup (2024) report reveals a global decline in well-being, with only 23% of employees engaged and 41% experiencing daily stress. This crisis is acute in Sub-Saharan Africa, where 48% report daily stress, and only 17% are thriving. In Ghana, the figures are particularly stark: 54% daily stress, 16% engagement, and just 24% flourishing. These statistics reveal an urgent need for organizations, especially high-stakes institutions like the military, to shift from merely preventing distress to actively promoting high-level well-being, or “workplace flourishing,” where employees feel good and function effectively (Seligman, 2011). For the Ghana Armed Forces (GAF), whose personnel face extraordinary stress and whose effectiveness is vital to national security, this is not a peripheral issue but a critical operational concern.

### 1.1 Problem statement

The Ghana Armed Forces (GAF) are a cornerstone of national security, relying entirely on the capability and well-being of their personnel. These individuals operate under extreme stress, requiring immense physical, emotional, and psychological resilience. However, recent data indicates a severe well-being crisis within the Ghanaian workforce. Gallup’s 2024 report shows only 24% of employees in Ghana are flourishing—a rate well below the global average. This decline poses a direct threat to the operational effectiveness of critical institutions like the military. While the negative impact of stress is well-documented, research on actively promoting high-level well-being—workplace flourishing—is scarce, especially in Sub-Saharan Africa and the unique context of the military.

Research specifically focusing on high-level well-being (workplace flourishing) in contexts like Sub-Saharan Africa, and more precisely Ghana, remains scarce. Previous research on well-being has often focused on isolated dimensions, such as the psychological, social, or physical, treated separately rather than as part of a holistic system (Bannay and Hadi, 2021; Lee, 2022; Karlou et al., 2019). This siloed approach has been identified as a key limitation, prompting calls for a more integrated research agenda that examines how these dimensions interact to produce high-level well-being, or flourishing (Inceoglu et al., 2018; Gable and Haidt, 2005). In response, recent scholarship and systematic reviews have advocated for a cohesive model that combines emotional, psychological, and social well-being, arguing that this reflects a more accurate and comprehensive view of employee well-being (Fabricio et al., 2022; A’yuninnisa et al., 2023; Seligman, 2011). This study seeks to answer that call by investigating flourishing as a unified construct. Two evidence-based studies that specifically explored workplace flourishing within the context of work: a systematic review spanning from 1960 to 2019 and another from 2020 to mid-2022, conducted by Fabricio et al. (2022) and A’yuninnisa et al. (2023). These studies unequivocally emphasized that research on workplace or job flourishing is still in its infancy, resulting in a scarcity of scholarly work in this area since most studies have looked at different forms of well-being separately and not high levels of well-being based on several criteria simultaneously. Consequently, these scholars strongly recommended that the international scientific and research community delve deeper into this theme, thereby justifying the ongoing scholarly attention it deserves. This present study seeks to fill this knowledge gap in the Ghanaian context.

Furthermore, workplace flourishing to a large extent significantly improved task performance. Employees who flourish are more engaged, motivated, and persistent, leading to enhanced job performance (Cerfontyne, 2020; Redelinghuys et al., 2019; van Rensburg et al., 2018). For example, Redelinghuys et al. (2019) found that flourishing significantly impacts IT professionals’ performance, while Cerfontyne (2020) noted that flourishing dimensions predict in-role performance in the public sector. However, other studies, such as Ramsay (2019), found no significant correlation between flourishing and performance in different contexts. These mixed findings indicate that more research is needed, particularly in the unique setting of the Ghanaian military.

Research on workplace flourishing and task performance has been extensively explored in Asian and European countries, including China, Indonesia, Pakistan, Malaysia, Switzerland, and the United States (Ugwoke et al., 2023; Kleynhans et al., 2022; Girei et al., 2022; Hayes, 2018; Cerfontyne, 2020). In contrast, there is a notable lack of studies focusing on these constructs within African contexts. This paper seeks to address three major gaps, namely contextual, sectoral, and issue-based gaps, thus establishing the lack of research in (1) flourishing as a holistic construct, (2) African contexts, and (3) military organizations. This paper investigates the nexus between workplace flourishing and task performance among military personnel (GAF) in the Ghanaian environment.

## 2 Literature review

### 2.1 Workplace flourishing

Workplace flourishing, also termed as “flourishing-at-work,” is characterized by a positive and fulfilling environment that nurtures the collective “emotional, psychological, and social well-being” of employees. This environment enables individuals to flourish and reach their optimal functioning levels, supported by a healthy organizational context (Kundi et al., 2021; Redelinghuys et al., 2019; Ariza-Montes et al., 2018).

From an employee perspective, workplace flourishing represents a transition from merely functioning to truly flourishing. Employees who flourish experience high levels of well-being, including positive emotions, a high degree of work engagement, job satisfaction, a sense of purpose, autonomy, social acceptance, integration and positive relationships. This flourishing state is essential for fostering resilience, motivation, and engagement, ultimately leading to greater personal fulfillment and professional success (Ho and Chan, 2022; Diedericks, 2018; Martin, 2021; Naim and Ozyilmaz, 2022). For organizations, the benefits of workplace flourishing are equally significant. A flourishing workforce drives higher levels of performance, productivity, and organizational reputation. Flourishing employees are more engaged, motivated, and committed, which directly translates into enhanced efficiency and effectiveness within the organization. Thus, the positive outcomes of flourishing extend beyond individual success, contributing to the overall growth and sustainability of the organization (Hunsaker and Ding, 2022; Wissing et al., 2021; Pleeging et al., 2021; Erum et al., 2020). In essence, workplace flourishing creates a positive feedback loop that benefits both employees and organizations.

#### 2.1.1 Operationalizing the workplace flourishing construct

For this study, workplace flourishing is conceptualized and operationalized as a second-order latent construct comprised of three interrelated first-order dimensions: emotional, psychological, and social well-being. This tripartite model is measured using the Flourishing at Work Scale (FAWS) developed by Rothmann (2013) and validated in subsequent research (e.g., Rautenbach, 2015). This operationalization aligns with the holistic well-being perspective advocated by Seligman (2011) and addresses recent calls for a more integrated approach to well-being research (Fabricio et al., 2022; A’yuninnisa et al., 2023). The three dimensions are defined and operationalized as follows:

- Emotional Well-being constitutes the affective component of flourishing, characterized by the frequent experience of positive emotions toward one’s work. This dimension is measured through items capturing feelings of happiness, excitement, energy, and contentment in the workplace (e.g., “I feel happy at work,” “I feel energetic at work”).
- Psychological Well-being captures the cognitive and developmental aspects of work life, encompassing a sense of purpose, autonomy, mastery, and growth. It is measured through items that assess perceived competence, autonomy, engagement, and the sense that one is realizing their potential (e.g., “I am engaged in my work,” “I see myself as capable at work”).
- Social Well-being pertains to the interpersonal dimension, focusing on the quality of an individual’s integration and relationships within the workplace community. It is measured through items evaluating feelings of belongingness, social acceptance, positive relationships with colleagues, and the sense of contributing to a community (e.g., “I feel I belong at work,” “My relationships at work are positive”).

The FAWS utilizes a 5-point frequency response format (from “never” to “everyday”) to capture the prevalence of these experiences. By synthesizing these three dimensions, the scale provides a comprehensive metric for an employee’s holistic state of flourishing, moving beyond the mere absence of distress to actively gauge high-level well-being. The adoption of Rautenbach and Rothmann’s (2017b) conceptualization of flourishing at work was used in this study.

### 2.2 Task performance

Task performance plays a pivotal role in enhancing employee productivity and driving organizational success. It involves the extent to which employees effectively carry out their job responsibilities, demonstrating their ability to skillfully meet expected standards (Oyong et al., 2023). Task performance reflects both the quality and quantity of work produced by individuals and teams, emphasizing how their efforts shape organizational outcomes (Katebi et al., 2022). This concept extends beyond mere task completion, encompassing the duties and expectations that define the contractual relationship between employers and employees, as well as managers and subordinates (Li et al., 2022; Uchhal and Solkhe, 2017). Scholars like Robbins and Judge (2022) and Pradhan and Jena (2016) argue that proficiently executing these responsibilities forms the technical backbone of any organization. Consequently, task performance is a critical determinant of organizational effectiveness, often reflected in a company’s financial results, where the quality and efficiency of employees’ work directly impact overall income and business outcomes (Lee et al., 2023). Therefore, high levels of task performance bring significant benefits, including enhanced productivity and operational efficiency (Ichdan, 2024; Mojza and Sonnentag, 2009), heightened employee engagement and satisfaction, and reduced turnover rates (Naqshbandi et al., 2024; Saks and Gruman, 2018). Additionally, when employees perform well, it not only sparks creativity and innovation as they tackle problems and take initiative but also leads to better financial results, happier customers, and a more resilient organization (Amabile and Pratt, 2016).

### 2.3 Empirical studies on workplace flourishing and task performance

A growing body of empirical work suggests that flourishing employees tend to perform better in their roles. Empirical work suggests that flourishing employees tend to perform better in their roles. For instance, studies of IT professionals and public sector workers have found that psychological well-being and other flourishing dimensions significantly predict task performance (Cerfontyne, 2020; Redelinghuys et al., 2019; van Rensburg et al., 2018). Also, van Rensburg et al. (2018), who, applying the Job Demands-Resources (JD-R) model, identified job resources such as role clarity and advancement as crucial enablers of flourishing, which in turn positively influenced academic employees’ performance. These studies converge on the view that flourishing, particularly when supported by enabling environments, enhances job performance. Other scholars support this perspective by highlighting the indirect mechanisms through which flourishing improves performance. Bahmani et al. (2016) found that flourishing influenced performance by boosting emotional, psychological, and social resources. Similarly, Rautenbach and Rothmann (2017a) argued that flourishing fosters reduced turnover intentions and heightened in-role performance, with implications for both employee retention and productivity. Further reinforcing this position, studies by Diedericks and Rothmann (2014), Sparks et al. (2001), and Lyubomirsky et al. (2005) emphasized that flourishing contributes to optimal individual functioning, job satisfaction, and positive affect, which collectively facilitate higher task performance. Lyubomirsky et al., in particular, proposed that flourishing enhances access to personal resources and emotions that act as enablers of successful work behavior.

Despite this overall positive narrative, not all empirical findings converge. Ramsay (2019), studying employees in a South African pharmaceutical company, found no significant correlation between flourishing and job performance. This divergence may be attributed to sector-specific demands; in high-regulation environments like pharmaceuticals, task performance may be shaped more by procedural compliance and external controls than by intrinsic well-being. Ramsay suggested that traditional performance metrics might not capture the nuanced effects of flourishing in such complex settings and recommended the use of industry-specific psychometric tools for better evaluation.

Taken together, the literature presents a largely affirmative case for the positive influence of workplace flourishing on task performance, while also highlighting exceptions that stress the importance of sectoral and contextual factors. The preponderance of evidence supports the view that flourishing employees are more productive, especially when psychological well-being is high and organizational resources are present to support autonomy, engagement, and clarity in role expectation. However, most of this research has been conducted in South Africa and other developed contexts, leaving a gap in understanding how flourishing operates in other regions, particularly in Sub-Saharan Africa. Notably, no known empirical studies have investigated the relationship between workplace flourishing and task performance in Ghana. This absence presents a significant gap, especially given the rising interest in employee well-being across African organizations and the need to contextualize psychological constructs within local cultural and institutional realities.

In light of the accumulated evidence and the identified contextual gaps, this study proposes to examine the flourishing–performance relationship in Ghana’s high-stakes organizational context, specifically the military healthcare setting. By doing so, it aims to contribute to the global conversation on workplace flourishing while filling a crucial empirical void in West Africa.

*H1: Workplace flourishing is positively associated with task performance.*

### 2.4 Theoretical underpinning: an integrated social exchange and job demands-resources perspective

This study is grounded in an integrated theoretical framework that combines Social Exchange Theory (SET) (Blau, 1964) and the Job Demands-Resources (JD-R) model (Bakker and Demerouti, 2007) to provide a comprehensive explanation for the relationship between workplace flourishing and task performance. While SET provides the fundamental motivational mechanism of reciprocity, the JD-R model elaborates the functional pathways through which workplace resources cultivate the well-being that is central to this exchange. Together, they offer a powerful lens for understanding this dynamic within the unique context of the Ghana Armed Forces (GAF).

Social Exchange Theory (SET) posits that human relationships are formed and sustained through the mutual exchange of valued resources, guided by a norm of reciprocity. Within the workplace, this translates to employees reciprocating beneficial treatment from their organization with greater effort, commitment, and performance (Cropanzano and Mitchell, 2005). Applying SET to this study, workplace flourishing is perceived as a valuable benefit or resource provided by the organizational environment. The experience of positive emotions (emotional well-being) generates a sense of obligation, motivating personnel to reciprocate through heightened task performance. Similarly, the resources gained from psychological well-being (e.g., autonomy, personal growth, a sense of purpose) and social well-being (e.g., positive relationships, team integration, belonging) are valued commodities in this exchange. Employees who receive these benefits are more likely to repay the organization with increased productivity and excellence in their roles.

The Job Demands-Resources (JD-R) model provides the critical link that explains *how* the organization provides the conditions for flourishing, thereby initiating the social exchange. As a dominant framework for understanding employee well-being (Granziera et al., 2021), the JD-R model categorizes all job characteristics into demands (aspects requiring sustained effort) and resources (aspects that aid goal achievement and stimulate growth). Central to this model is the motivational process, whereby job resources foster work engagement and other positive states, which in turn lead to enhanced performance (Bakker and Demerouti, 2017). The JD-R model allows us to specify the cognitive, affective, and motivational pathways through flourishing enhances task performance: (1) Affective Pathway- the emotional well-being dimension of flourishing—characterized by frequent experiences of happiness, energy, and contentment (e.g., “I feel happy at work”)—induces a positive affective state. According to the Broaden-and-Build Theory (Fredrickson, 2001), positive emotions broaden individuals’ thought-action repertoires, enhancing creativity and flexibility in problem-solving, while also building lasting personal resources like resilience, which is critical for performance in demanding military contexts.(2) Cognitive Pathway- the psychological well-being dimension—comprising engagement, autonomy, and a sense of mastery (e.g., “I am engaged in my work”)—reduces the cognitive load associated with stress and alienation. This conserves finite cognitive resources, allowing personnel to maintain sharper focus, better information processing, and more efficient decision-making when executing their tasks (Binnewies et al., 2009). (3) Motivational Pathway- the social well-being dimension—encompassing belongingness and positive relationships (e.g., “I feel I belong at work”)—fulfills the fundamental human need for relatedness (Deci and Ryan, 2011). This, combined with the purpose and autonomy from psychological well-being, fosters intrinsic motivation and a deep sense of work engagement. Engaged and motivated employees naturally exert greater effort, persist longer in the face of challenges, and are more committed to achieving high-quality outcomes in their core duties.

In this integrated view, the dimensions of workplace flourishing are directly fueled by job resources. Psychological well-being (e.g., engagement, autonomy, mastery) is cultivated through resources like role clarity and opportunities for advancement. Social well-being (e.g., belonging, positive relationships) is built through supportive leadership and team cohesion. Emotional well-being (e.g., happiness, energy) arises from a resource-rich environment that mitigates excessive job demands. Thus, the JD-R model establishes workplace flourishing not as an abstract concept, but as the direct outcome of the resources provided by the military institution. This integration resolves the theoretical gap noted by the reviewer. Also, the JD-R model explains the *functional mechanism* (resources → flourishing → performance), while SET provides the *relational motive* (the norm of reciprocity that obliges employees to repay the organization with performance). This is particularly salient in a military context, where loyalty and reciprocal obligation are core cultural tenets. The GAF, by providing job resources that cultivate flourishing, initiates a social exchange. Personnel then reciprocate this investment by channeling their flourishing—their energy, engagement, and commitment—into exemplary task performance, thereby enhancing the operational effectiveness of the force. In essence, SET provides the overarching “why” of the relationship (reciprocity), and the JD-R model elaborates the “how” (resource provision leading to well-being). This framework posits that the significant positive relationship between workplace flourishing and task performance exists because flourishing represents both a state of high motivational energy *and* a benefit that creates a debt of reciprocity, which is paid through enhanced performance.

## 3 Methodology

A quantitative cross-sectional design was used to measure specific characteristics within the sample and analyze information from a structured data collection process (Davis and Sandifer-Stech, 2006). For this research, the population consisted of military personnel, including Commissioned Officers and Non-Commissioned Officers (NCOs), serving in the Ghana Armed Forces (GAF), specifically those stationed at the 37 Military Hospital in the Greater Accra Region. The targeted military personnel comprised ranks ranging from Second Lieutenant (2/Lt) to Major (Maj) among Commissioned Officers, and from Warrant Officers to Privates among NCOs, including equivalents in other Arms of Service. Furthermore, the choice of facility for this research was influenced by several key factors. Firstly, the 37 Military Hospital is the largest military medical facility in Ghana, featuring a Tri-Service composition that includes personnel from the Army, Navy, and Air Force. This unique setting provided an unparalleled research environment, attracting a diverse population of military personnel, including both combat troops and support staff. Out of 619 total population, a sample size of 370 personnel was determined using Miller and Brewer’s (2003) formula. Stratified sampling technique was used to ensure adequate representation within each stratum —Army, Navy, and Air Force—within the GAF. In terms of inclusion and exclusion criteria, the study targeted personnel in the armed forces across various units and departments within the Forces. The inclusion criteria established for participants were as follows. First, participants had to be currently serving members of the Ghana Armed Forces to ensure relevance to the study context. Second, participants were required to be actively engaged in their military responsibilities within the Ghana Armed Forces during the time of data collection to capture their current experiences. Third, the study included participants from the ranks of Second Lieutenant to Major (commissioned officers) and Non-Commissioned Officers (NCOs), as well as their equivalents in other branches of service. The exclusion criteria established for the study were as follows. First, senior officers, defined as those holding ranks from Lieutenant Colonel and above, including Generals, Brigadier Generals, Commodores, Admirals, Colonels, Marshals of the Air Force, and Air Commodores, as well as their equivalents in other services, were excluded from participation. Second, non-military personnel, including civilians or individuals not directly affiliated with the Ghana Armed Forces, were also excluded. This decision aimed to maintain the homogeneity of the sample and ensure the unique military context was preserved, thereby facilitating the attainment of the research objectives.

Standardized questionnaires were adopted, namely the Flourishing at Work Scale (FAWS adopted from Rothmann (2013) and used by Rautenbach (2015) which operationalizes the multidimensional construct as described in section 2.1.1. The 5-point response format from “never, once or twice, unsure, few times and everyday”). Task performance was assessed using Williams and Anderson's (1991) in-role performance scale (IPS). It is a seven-item scale with a 5-point Likert-type agreement scale (1 = strongly disagree; 5 strongly agree). In terms of reliability test, Cronbach’s alpha values obtained for each scale of reliability were as follows: Workplace Flourishing Scale: 0.950 and Task Performance Scale: 0.833.

Given that data for both the predictor and outcome variables were collected from the same source via a self-report questionnaire, the potential for common method variance (CMV) was considered. To statistically assess this risk, this paper employed Harman’s single-factor test (Podsakoff et al., 2003). An exploratory factor analysis including all items from the Workplace Flourishing and Task Performance scales was conducted. The unrotated factor solution revealed multiple factors with eigenvalues greater than 1, and no single factor emerged that accounted for the majority of the covariance (the largest factor explained less than 50% of the total variance). This result indicates that CMV is not a likely contaminant of the observed relationships in this study. In terms of Structural equation modeling [SEM analysis, the hypothesized relationship between workplace flourishing and task performance was tested using Structural Equation Modeling (SEM) in Amos version 21]. The maximum likelihood estimation method was employed. The model consisted of workplace flourishing as the predictor variable and task performance as the outcome variable.

This study was conducted in strict accordance with ethical principles for research involving human participants. Before data collection commenced, ethical approval was obtained from two independent bodies: the Ethics Committee for Humanities at the University of Ghana (March 2024) and the Institutional Review Board (IRB) of the 37 Military Hospital (May 2024). Informed consent was obtained from all participants before their involvement in the study. The consent process clarified the study’s purpose, assured participants of their anonymity and confidentiality, and emphasized the voluntary nature of participation, including the right to withdraw at any time without consequence. To protect participant privacy, all data were collected and handled with strict confidentiality. No personally identifiable information was recorded on the questionnaires. All data were stored securely and were accessible only to the research team, with all identifying information scheduled for deletion after the study’s completion.

## 4 Results and discussion of findings

### 4.1 Descriptive statistics - demographics

The analysis revealed that 57% of respondents were between 30 to 39 years, 30% were between 18 to 29 years, and 14% were between 40 to 49 years. In terms of gender, 66% of the respondents were male, while 34% were female, reflecting the gender composition of the workforce. The type of force respondents belonged to was also analyzed, with 47% from the Army, and 26% each from the Navy and Air Force (Table 1).

**Table 1:** Summary of demographic characteristics.

Characteristic	Category	Frequency	Percentage (%)
Age	18–29 years	87	29.8
	30–39 years	165	56.5
	40–49 years	40	13.7
Gender	Male	191	65.5
	Female	101	34.6
Marital Status	Married	83	28.4
	Single	182	62.3
	Divorce/widowed	27	9.2
Educational Level	SSS/SHS	61	20.9
	Voc./Tech	45	15.4
	Tertiary	147	50.3
	Post-Graduate	39	13.4
Work Experience	Less than a year	15	5.1
	1–5 years	133	45.5
	6–10 years	98	33.6
	11–15 years	33	11.3
	16–20 years	12	4.1
	21 years and above	1	0.3
Type of Force	Army	138	47.3
	Navy	77	26.4
	Airforce	77	26.4
Total		292	100

Source: Field data (2024).

### 4.2 Correlation analysis

The relationship between workplace flourishing and employee task performance was also found to be significant (*r* = 0.486, *p* < 0.01) (see Table 2). This strong correlation implies that employees who flourish in their work environment not only experience personal well-being but also show higher levels of job performance, underscoring the link between a positive work atmosphere and productivity. Lastly, the analysis explored the potential impact of demographic variables—such as age, gender, marital status, education, experience, and type of work setting—on workplace flourishing and employee task performance. However, the findings indicated that none of these demographic factors had a significant relationship with either workplace flourishing or employee task performance.

**Table 2:** Descriptive statistics and correlation analysis.

#	Variable	Mean/Median	SD	1	2	3	4	5	6	7	8	9	10	11	12
1	Age	34.5	0.641	1											
2	Gender	1.35	0.476	−0.020	1										
3	Marital Status	1.81	0.584	−0.046	−0.032	1									
4	Education	5.56	0.967	0.285**	−0.155**	0.015	1								
5	Experience	2.65	0.921	0.439**	−0.034	0.021	0.424**	1							
6	Type of Force	1.79	0.834	−0.018	0.053	0.030	−0.029	−016	1						
7	WPF	5.607	0.942	0.079	0.024	−0.037	−0.071	0.064	0.062	0.137*	0.260**	0.242**	0.347**	1	
8	ETP	5.289	0.998	−0.073	−0.089	0.031	−0.061	−0.011	−0.170**	0.366**	0.436**	0.386**	0.341**	0.486**	1

Source: Field Data (2024), WPF=Workplace flourishing, ETP = Employee Task performance.

**Figure 1:**
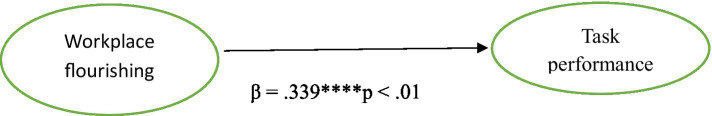
Relationship between workplace flourishing and task performance. *β* = 0.339****p < 0.01.

This objective sought to examine the relationship between workplace flourishing and employee task performance among the Ghana Armed Forces. This objective was measured by developing the hypothesis (H1), which stated that workplace flourishing has a positive relationship with task performance. This hypothesis was tested using SEM analysis via Amos (Version 21), workplace flourishing being the predictor variable, while task performance was the predicted variable. The overall fit of the model was assessed using standard goodness-of-fit indices. The results indicated an acceptable fit to the data: χ^2^/df = 2.406, Comparative Fit Index (CFI) = 0.936, Incremental Fit Index (IFI) = 0.940, Root Mean Square Error of Approximation (RMSEA) = 0.070, and Standardized Root Mean Square Residual (SRMR) = 0.075. These values all fall within the acceptable thresholds recommended by Hu and Bentler (1999) and Hair et al. (2010), indicating that the model is a good representation of the observed data. Furthermore, as shown in Table 3, the SEM analysis supported the hypothesis, revealing a significant positive predictive relationship between workplace flourishing and task performance (*β = 0*.339, *SE = 0*.054, *p < 0*.01). Figure 1 illustrates this path model.

**Table 3:** Direct pathway relationships assessment based on the study objective.

Hypothesis	Path	Estimate	S. E	C. R	*p*-value	Interpretation
H1	WPF → ETP	0.339	0.054	6.779	0.001	Significant (*Supported*)

This finding implies that workplace flourishing significantly and positively predicts task performance of the Ghana Armed Forces. Therefore, H1 was accepted. The significant positive association suggests that as workplace flourishing increases, in terms of feeling happy, excitement, energy, and belongingness, they tend to be more engaged with their tasks toward a common goal (task performance increases). This implies that a supportive and fulfilling work environment can enhance the effectiveness of military personnel in their roles. This outcome emphasizes the importance of promoting a flourishing environment within military settings. When personnel experience higher levels of well-being and fulfillment at work, it can lead to improved performance on tasks critical to the military. Further analysis investigated potential differences across the military hierarchy; participants’ sample (*N* = 292) were grouped into three categories: Junior Enlisted (*n* = 137), Non-Commissioned Officers (NCOs) (*n* = 99), and Commissioned Officers (*n* = 56). A one-way analysis of variance (ANOVA) revealed no statistically significant mean differences across Rank Groups for workplace flourishing (*F* (2, 289) = 0.65, *p* = 0.52) or task performance (*F* (2, 289) = 0.40, *p* = 0.67). Each table shows that average scores do not differ significantly by rank group. This suggests that the experience of flourishing and self-reported performance levels are consistent across hierarchical levels within the Ghana Armed Forces, thus implying that the benefits of a flourishing environment are universal across these hierarchical tiers (Table 4).

**Table 4:** Workplace flourishing ANOVA table.

Source	Sum of squares	df	Mean square	*F*	*p-*value
Between groups	0.65	2	0.33	0.65	0.52
Within groups	145.50	289	0.50		
Total	146.15	291			

This study provides robust empirical evidence that workplace flourishing is a significant positive predictor of task performance among military personnel in the Ghana Armed Forces (GAF). This finding aligns with a body of work conducted primarily in Western contexts (e.g., Redelinghuys et al., 2019; Cerfontyne, 2020, van Rensburg et al. 2018) and confirms that the positive relationship between holistic well-being and performance holds even within the unique, high-stakes environment of a military organization in Sub-Saharan Africa. However, the existing literature also presents some contradictory evidence. For example, Ramsay (2019) found no significant correlation between flourishing and job performance in a pharmaceutical company context. This highlights the complexity of the relationship and suggests that contextual factors may influence how workplace flourishing influences performance in different industries. The finding that flourishing drives performance is not self-evident; it requires explanation grounded in psychological theory. Our integrated Job Demands-Resources (JD-R) and Social Exchange Theory (SET) framework provides the necessary lens to interrogate this mechanism (Table 5).

**Table 5:** Task Performance ANOVA Table.

Source	Sum of squares	df	Mean square	*F*	*p*-value
Between groups	0.50	2	0.25	0.40	0.67
Within groups	180.75	289	0.63		
Total	181.25	291			

**Figure 2:**
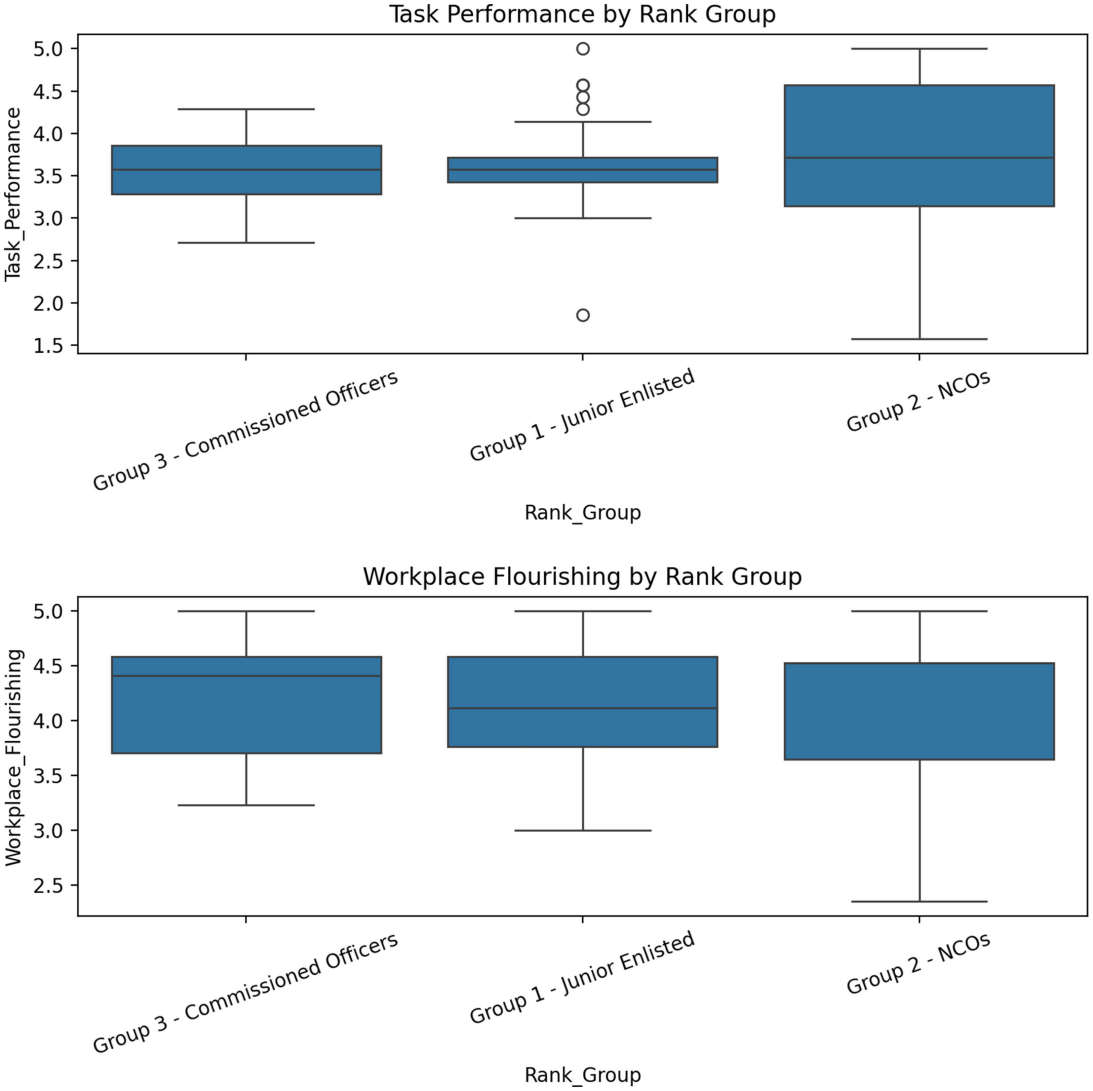
Path model for workplace flourishing and task performance.

From a theoretical perspective, that is JD-R perspective, the results suggest that the dimensions of flourishing—emotional, psychological, and social well-being—are not merely outcomes but function as critical personal resources. Specifically emotional well-being (e.g., positive affect, energy) likely broadens cognitive and behavioral repertoires, allowing personnel to be more creative and resilient problem-solvers (Fredrickson, 2001). Psychological well-being (e.g., autonomy, mastery, purpose) provides the cognitive drive and sense of efficacy needed to persist with complex tasks. Social well-being (e.g., belonging, positive relationships) provides the collaborative support necessary for effective task completion in a team-based environment. Concurrently, Social Exchange Theory (SET) explains the relational motive behind this resource investment. Also, theory posits that positive employee experiences—such as emotional and psychological well-being—act as benefits that employees feel compelled to reciprocate with increased effort and performance. Within the GAF’s collectivist culture, the experience of flourishing is likely perceived not just as a personal state but as a benefit conferred by the military institution. This perception creates a powerful sense of obligation and a debt of reciprocity. Personnel repay this debt by investing their personal resources (their energy, drive, and collaborative spirit) back into the organization through heightened task performance. This creates a virtuous cycle: the organization provides conditions for flourishing, and employees reciprocate with performance, which justifies further investment in well-being. Therefore, present findings do not merely support one theory over another; they demonstrate that JD-R and SET are complementary. The JD-R model explains the functional pathway (resources → flourishing → performance), while SET explains the relational catalyst (reciprocity) that intensifies this pathway within a structured, duty-bound organization like the military. This integration challenges a simplistic view of well-being as a mere “feel-good” factor and re-frames it as a strategic resource in a social exchange (Figure 2).

In terms of practical implications, military organizations should prioritize initiatives that promote workplace flourishing, as this can enhance the effectiveness of personnel in critical roles. By focusing on well-being and fulfillment, military leaders can not only benefit individual soldiers but also improve overall operational efficiency. This study indicates that investing in the social, psychological, and emotional well-being of military personnel can lead to a more effective and resilient force, ultimately benefiting the organization and its mission. In a broader context, these findings reinforce the notion that workplace flourishing is essential in high-pressure environments like the military. By cultivating a positive workplace culture, military leaders can enhance engagement and performance, which are vital for success in demanding situations. Ultimately, the current study not only corroborates previous research that underscores the beneficial effects of workplace flourishing on task performance but also emphasizes the relevance of JD-R and SET in explaining these outcomes. The findings suggest that fostering a flourishing environment within military organizations is crucial for enhancing personnel effectiveness. Furthermore, as this research is one of the few conducted in Ghana, it contributes valuable insights to the academic landscape, highlighting the need for further exploration of these dynamics in varied contexts. Lastly, the study encourages organizations to consider the broader implications of employee well-being, underscoring that a flourishing workforce can lead to improved performance outcomes across various sectors.

## 5 Summary, implications, limitations, and recommendations for future research

This study investigated the relationship between workplace flourishing and task performance in the military, specifically within the Ghana Armed Forces (GAF). A clear, positive relationship emerged between flourishing and task performance. Soldiers who felt psychologically and emotionally well-adjusted were more engaged, motivated, and productive. This reinforces the argument that workplace well-being is not a peripheral issue but a direct contributor to mission success, especially in environments as demanding as the military. This study addresses a significant gap in the literature regarding workplace flourishing and task performance within military contexts. It offers valuable insights into the emerging concept of workplace flourishing in professional settings. The current study also sheds new light on the military context, which has been less explored in previous research. This study contributes to the theoretical discourse on well-being, specifically workplace flourishing and task performance, by extending the applicability of Social Exchange Theory (SET) and JD-R model into the military context.

In terms of practical implications, the significant positive link between workplace flourishing and enhanced task performance, established by this study, provides compelling evidence base for the Ghana Armed Forces (GAF) to strategically reevaluate its approach to personnel development. Rather than treating well-being as a peripheral concern, these findings argue for its formal integration into the core of human resource policy and leadership doctrine. Moving beyond ad-hoc initiatives, the GAF should consider institutionalizing these practices to systematically build a more effective force. The three dimensions of flourishing offer a concrete framework for this integration (1). Fostering Psychological Well-Being-Leadership curricula at institutions like the Ghana Armed Forces Command and Staff College (GAFCSC) should be updated to emphasize autonomy-supportive command techniques. Training commanders to provide clear strategic intent while delegating tactical execution—where operationally viable—can directly cultivate the purpose, mastery, and ownership that underpin psychological resilience. To incentivize this, performance appraisal systems for unit leaders could formally incorporate metrics related to subordinate well-being and climate surveys, tethering leadership success directly to the health of their teams. (3) Building Social Well-being- The GAF could significantly benefit from mandating structured, cross-rank mentorship programs. Pairing junior enlisted personnel and officers with seasoned NCOs and senior officers would fortify unit cohesion and provide essential social support networks that operate outside the formal chain of command. Furthermore, commanding officers should be evaluated on their proactive efforts to facilitate team-building, recognizing these activities not as optional diversions but as critical investments in unit readiness and operational trust. (3) Encouraging Emotional Well-being- A systematic approach to recognition is required. The establishment of formal, transparent programs that regularly acknowledge individual and unit achievements—through a mix of awards, commendations, and public recognition—is crucial for validating effort and sustaining the positive emotions that drive motivation. The routine administration of command climate surveys, with results actionable at the highest levels, would ensure accountability and provide data to continuously refine these efforts. Therefore, this study proposes that these recommendations be piloted as a cohesive human performance optimization framework. By deliberately engineering a flourishing environment through these policy-level changes, the GAF can leverage the findings of this study to directly enhance operational capability. This strategic pivot redefines investment in personnel well-being from a mere administrative cost into a demonstrable cornerstone of national security.

Notwithstanding its contributions, this study has several limitations. First, the sample was confined to military personnel stationed at the 37 Military hospital Security Area of the Ghana Armed Forces, limiting the generalizability of the findings to other military settings. Also, concentrate on the quantitative approach and focus on commissioned and non-commissioned officers, excluding top senior commissioned officers. To address the above limitation, future research should expand the scope by including military personnel from other bases or headquarters to capture a more diverse range of experiences. Incorporating top senior commissioned officers into the sample could offer a more comprehensive perspective on leadership. Furthermore, the cross-sectional design demonstrates the correlation and statistical prediction, but cannot definitively prove causality. Longitudinal studies are needed to untangle this temporal sequence. Reliance on self-reported performance data. While practical constraints in the military setting made objective or supervisor-rated data unfeasible, the paper acknowledges that this introduces the potential for inflation of the relationship due to common method variance. However, statistical checks (Harman’s test) suggest this is not a severe threat, and self-perceptions of performance are themselves meaningful. Also, the use of well-established, validated scales with high reliability, coupled with the strength of the significant results, provides confidence that the findings reflect a robust relationship between the constructs. Nevertheless, future research should strive to incorporate multi-source data, such as supervisor ratings of performance, to circumvent this potential bias. Also, extending research into other high-stress environments, such as police or security agencies, could help generalize findings across different sectors. Lastly, this study establishes a direct relationship between flourishing and performance in a novel context. However, future research should explore moderators (e.g., cultural/ institutional factors, branch of service, gender, tenure) to identify boundary conditions, and mediators (e.g., work engagement, self-efficacy) to uncover the precise psychological mechanisms underlying this relationship. Such work will provide a more nuanced model and offer leaders more precise interventions to enhance both well-being and operational effectiveness. In addition, two methodological limitations should be noted more explicitly. First, the study relied solely on self-reported measures of task performance. While validated scales with strong reliability were used and statistical checks (e.g., Harman’s single-factor test) suggested that common method variance was not a major concern, self-ratings may still be influenced by perceptual bias or social desirability. Future research should therefore consider triangulating self-reports with supervisor ratings, peer evaluations, or objective performance indicators to strengthen validity. Second, the use of a cross-sectional, correlational design limits the ability to make strong causal claims. Although the theoretical model is well-structured and predictive relationships were supported, the findings should be interpreted as tentative in terms of directionality. Longitudinal or experimental designs would be required to establish causal pathways between workplace flourishing and task performance.

This study offers notable strengths. It is one of the first to empirically determine the nexus between flourishing and performance in the Ghanaian military. For leadership within the GAF and similar institutions, this research provides a compelling evidence-based argument that investing in soldiers’ well-being is not a peripheral welfare program but a critical strategic investment in operational effectiveness. Commanders should be equipped and incentivized to act as architects of flourishing by providing the job resources—such as clear communication, autonomy where possible, and fostering unit cohesion—that trigger the positive cycle of well-being and performance. In conclusion, this study moves beyond establishing a direct link to propose a theoretically-grounded mechanism for how and why flourishing enhances performance in a military context. By integrating the JD-R and SET frameworks, this paper offers a model that can guide future research and leadership practice, affirming that the cultivation of victory begins with the cultivation of a flourishing force.

## Data Availability

The raw data supporting the conclusions of this article will be made available by the authors, without undue reservation.
